# (Nitrito-κ^2^*O*:*O*′)bis­[tris­(4-fluoro­phen­yl)phosphine-κ*P*]silver(I)

**DOI:** 10.1107/S2414314625002962

**Published:** 2025-04-08

**Authors:** Frederick P. Malan, Kariska Potgieter, Reinout Meijboom

**Affiliations:** a Department of Chemistry, University of Pretoria, Lynnwood Road, Hatfield, Pretoria, 0002, South Africa; b Department of Chemical Sciences, University of Johannesburg, PO Box 524, Auckland Park, 2006, Johannesburg, South Africa; Vienna University of Technology, Austria

**Keywords:** silver(I) complex, tris-4-fluoro­phenyl phosphine, nitrite, crystal structure

## Abstract

The mol­ecular structure of the title Ag^I^ complex, [Ag(NO_2_)(C_18_H_12_F_3_P)_2_], features a distorted tetra­hedral geometry about the central Ag^I^ atom, with a total range of bond angles spanning from 49.80 (5) to 114.92 (1)°. The distortion arises primarily due to the small bite angle [49.80 (5)°] of the nitrito ligand. The compound crystallizes with one mol­ecule in the asymmetric unit, in the space group *P*2_1_/*n*, with *Z* = 4 and *Z*′ = 1.

## Abstract 2

The significant structural feature in the title Ag^I^ complex is the small bite angle of the nitrito ligand [49.80 (5)°], which influences the overall geometry.

## Graphical abstract

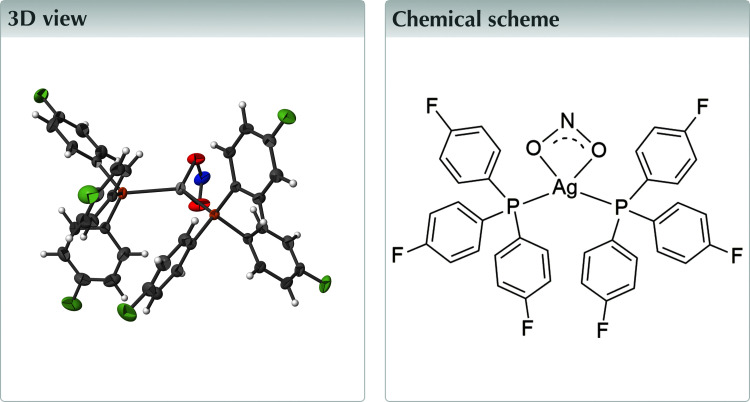

## Structure description

Silver(I) phosphine complexes exhibit significant anti­microbial, anti­bacterial, and anti­cancer activity (Potgieter *et al.*, 2016 ▸). Solid-state characterization, particularly single-crystal X-ray diffraction, is crucial for understanding structural features relevant to their function (Malan *et al.*, 2022*a* ▸).

**Figure 1:**
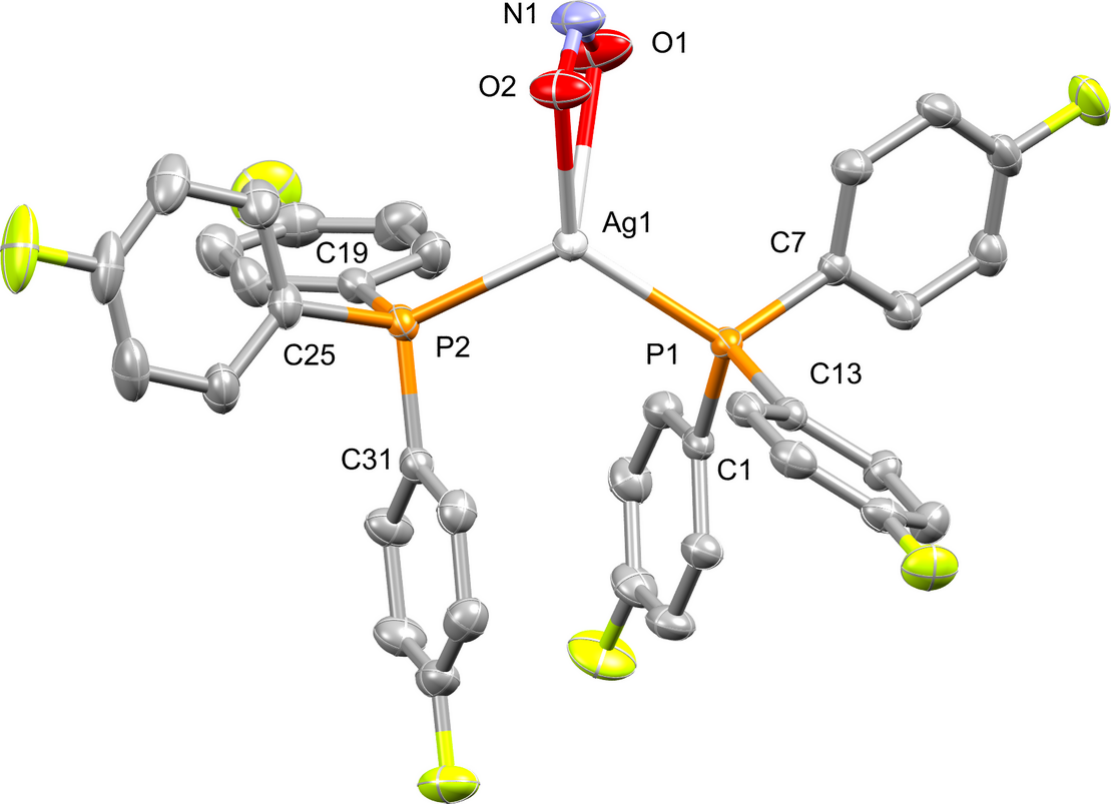
The mol­ecular structure of the title compound showing displacement ellipsoids at the 50% probability level. Hydrogen atoms are omitted for clarity.

Fig. 1 ▸ shows the mol­ecular structure of the title complex, [Ag(NO_2_)(C_18_H_12_F_3_P)_2_]. A distorted tetra­hedral coordination environment is observed around the central silver(I) atom, which comprises of a bidentate nitrito ligand [O1—Ag1—O2 = 49.80 (5)°, Ag1—O1 = 2.5638 (14) Å, Ag1—O2 = 2.3379 (13) Å], and two tris-4-fluoro­phenyl­phosphine ligands [P1—Ag1—P2 = 114.924 (14)°, Ag1—P1 = 2.4457 (4) Å, Ag1—P2 = 2.4680 (4) Å]. The *ipso*-aryl carbon atoms of each of the phosphine ligands appear in a near-staggered fashion when viewed down the P1—Ag1—P2 plane [C1—P1—P2—C19 = −60.97 (14)°, C7—P1—P2—C25 = −118.91 (13)°]. The plane defined by atoms P1, Ag1, and P2 inter­sects the plane defined by Ag1, O1, and O2 at an angle of 80.3 (8)°. All other bond lengths and angles correspond with related complexes (Potgieter *et al.*, 2016 ▸; Malan *et al.*, 2022*b* ▸).

**Figure 2:**
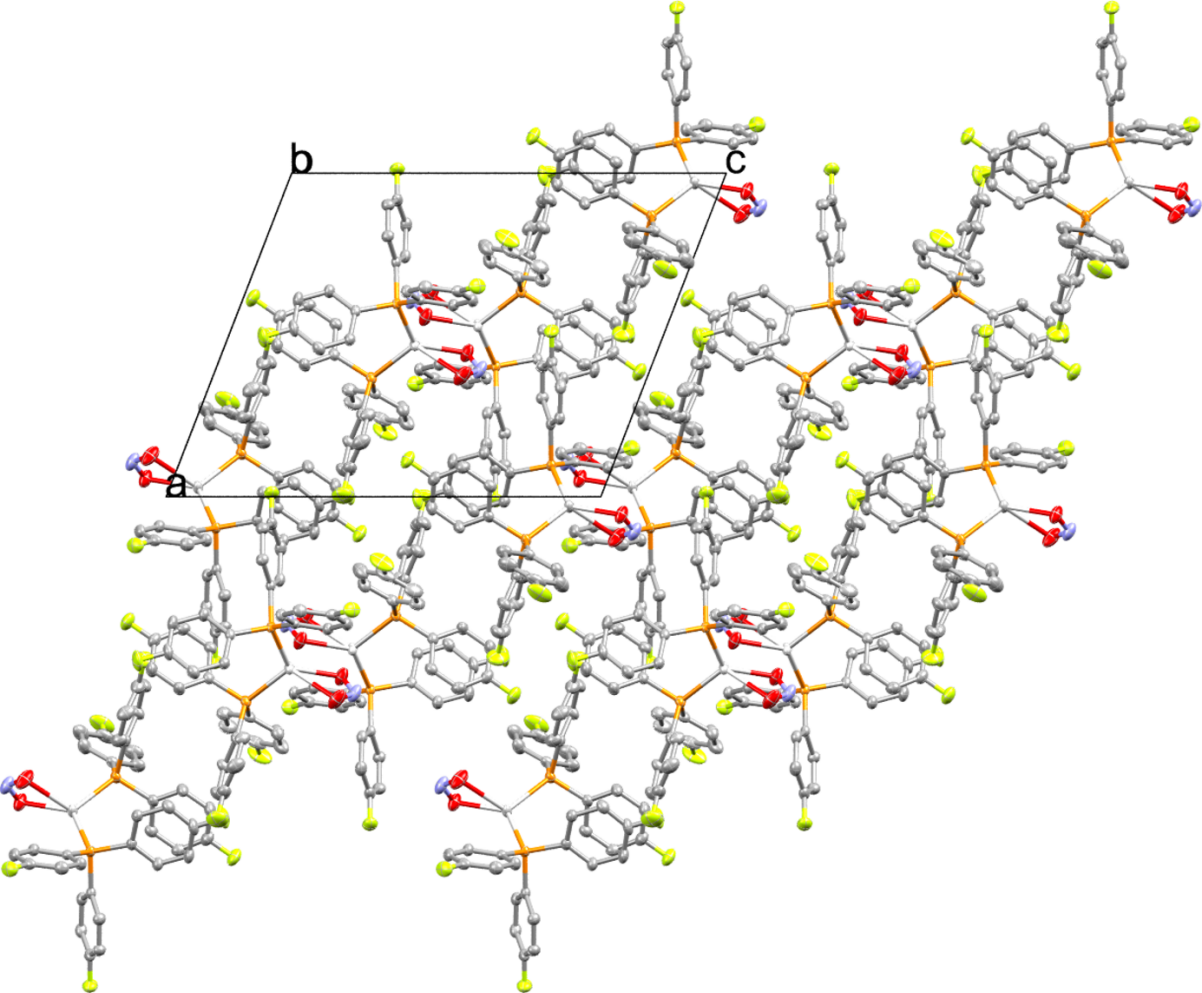
Packing diagram viewed along the *b* axis.

In the crystal packing (Fig. 2 ▸), the complexes arrange as discrete mol­ecular units with weak C—H⋯F hydrogen-bonding inter­actions (Table 1 ▸). Pairs of nearest-neighbour silver(I) atoms are separated by a distance of 4.337 Å, indicative of a very weak argentophilic inter­action (Schmidbaur & Schier, 2015 ▸). No other classical hydrogen bonding or significant close-packing motifs are observed.

## Synthesis and crystallization

A 1 mmol solution of silver nitrite was prepared in 10 ml aceto­nitrile and added to a solution of tris-4-fluoro­phenyl­phosphine (2 mmol) in 10 ml aceto­nitrile. The solution was stirred at 353 K, removed and left to slowly cool and crystallize.

## Refinement

Crystal data, data collection and structure refinement details are summarized in Table 2 ▸.

## Supplementary Material

Crystal structure: contains datablock(s) I. DOI: 10.1107/S2414314625002962/wm4226sup1.cif

Supporting information file. DOI: 10.1107/S2414314625002962/wm4226Isup3.cdx

Structure factors: contains datablock(s) I. DOI: 10.1107/S2414314625002962/wm4226Isup4.hkl

CCDC reference: 2440532

Additional supporting information:  crystallographic information; 3D view; checkCIF report

## Figures and Tables

**Table 1:** Hydrogen-bond geometry (Å, °)

*D*—H⋯*A*	*D*—H	H⋯*A*	*D*⋯*A*	*D*—H⋯*A*
C2—H2⋯F1	0.95	2.42	3.367 (2)	175
C35—H35⋯F6	0.95	2.40	3.203 (3)	143

**Table 2:** Experimental details

Crystal data	
Chemical formula	[Ag(NO_2_)(C_18_H_12_F_3_P)_2_]
*M*_r_	786.37
Crystal system, space group	Monoclinic, *P*2_1_/*n*
Temperature (K)	150
*a*, *b*, *c* (Å)	14.3478 (4), 13.5179 (3), 17.9803 (5)
β (°)	111.215 (3)
*V* (Å^3^)	3250.98 (16)
*Z*	4
Radiation type	Mo *K*α
μ (mm^−1^)	0.79
Crystal size (mm)	0.31 × 0.21 × 0.09

Data collection	
Diffractometer	XtaLAB Synergy R, DW system, HyPix
Absorption correction	Multi-scan (*CrysAlis PRO*; Rigaku OD, 2022 ▸)
*T*_min_, *T*_max_	0.481, 1.000
No. of measured, independent and observed [*I* > 2σ(*I*)] reflections	52869, 8729, 7454
*R*_int_	0.037
(sin θ/λ)_max_ (Å^−1^)	0.726

Refinement	
*R*[*F*^2^ > 2σ(*F*^2^)], *wR*(*F*^2^), *S*	0.026, 0.064, 1.05
No. of reflections	8729
No. of parameters	433
H-atom treatment	H-atom parameters constrained
Δρ_max_, Δρ_min_ (e Å^−3^)	0.35, −0.48

Computer programs: *CrysAlis PRO* (Rigaku OD, 2022 ▸), *SHELXT* (Sheldrick, 2015*a* ▸), *SHELXL* (Sheldrick, 2015*b* ▸), *OLEX2* (Dolomanov *et al.*, 2009 ▸) and *publCIF* (Westrip, 2010 ▸).

## full crystallographic data

### (Nitrito-κ2O:O')bis[tris(4-fluorophenyl)phosphine-κP]silver(I) Crystal data

**Table:**

[Ag(NO_2_)(C_18_H_12_F_3_P)_2_]	*F*(000) = 1576
*M**_r_* = 786.37	*D*_x_ = 1.607 Mg m^−^^3^
Monoclinic, *P*2_1_/*n*	Mo *K*α radiation, λ = 0.71073 Å
*a* = 14.3478 (4) Å	Cell parameters from 35295 reflections
*b* = 13.5179 (3) Å	θ = 2.7–31.1°
*c* = 17.9803 (5) Å	µ = 0.79 mm^−^^1^
β = 111.215 (3)°	*T* = 150 K
*V* = 3250.98 (16) Å^3^	Blade, colourless
*Z* = 4	0.31 × 0.21 × 0.09 mm

### (Nitrito-κ2O:O')bis[tris(4-fluorophenyl)phosphine-κP]silver(I) Data collection

**Table:**

XtaLAB Synergy R, DW system, HyPix diffractometer	8729 independent reflections
Radiation source: Rotating-anode X-ray tube, Rigaku (Mo) X-ray Source	7454 reflections with *I* > 2σ(*I*)
Mirror monochromator	*R*_int_ = 0.037
Detector resolution: 10.0000 pixels mm^-1^	θ_max_ = 31.1°, θ_min_ = 2.7°
ω scans	*h* = −18→20
Absorption correction: multi-scan (CrysAlisPro; Rigaku OD, 2022)	*k* = −19→19
*T*_min_ = 0.481, *T*_max_ = 1.000	*l* = −24→23
52869 measured reflections	

### (Nitrito-κ2O:O')bis[tris(4-fluorophenyl)phosphine-κP]silver(I) Refinement

**Table:**

Refinement on *F*^2^	Primary atom site location: dual
Least-squares matrix: full	Hydrogen site location: inferred from neighbouring sites
*R*[*F*^2^ > 2σ(*F*^2^)] = 0.026	H-atom parameters constrained
*wR*(*F*^2^) = 0.064	*w* = 1/[σ^2^(*F*_o_^2^) + (0.0247*P*)^2^ + 1.7214*P*] where *P* = (*F*_o_^2^ + 2*F*_c_^2^)/3
*S* = 1.05	(Δ/σ)_max_ = 0.003
8729 reflections	Δρ_max_ = 0.35 e Å^−^^3^
433 parameters	Δρ_min_ = −0.48 e Å^−^^3^
0 restraints	

### (Nitrito-κ2O:O')bis[tris(4-fluorophenyl)phosphine-κP]silver(I) Special details

**Table:**

Geometry. All esds (except the esd in the dihedral angle between two l.s. planes) are estimated using the full covariance matrix. The cell esds are taken into account individually in the estimation of esds in distances, angles and torsion angles; correlations between esds in cell parameters are only used when they are defined by crystal symmetry. An approximate (isotropic) treatment of cell esds is used for estimating esds involving l.s. planes.

### (Nitrito-κ2O:O')bis[tris(4-fluorophenyl)phosphine-κP]silver(I) Fractional atomic coordinates and isotropic or equivalent isotropic displacement parameters (Å^2^)

**Table:**

	*x*	*y*	*z*	*U*_iso_*/*U*_eq_	
Ag1	0.52949 (2)	0.37505 (2)	0.43613 (2)	0.02553 (4)	
P1	0.40331 (3)	0.25635 (3)	0.35813 (2)	0.01994 (8)	
P2	0.63301 (3)	0.44659 (3)	0.36593 (2)	0.02370 (9)	
F2	−0.01153 (8)	0.40981 (9)	0.23905 (7)	0.0405 (3)	
F1	0.34956 (9)	−0.10257 (9)	0.52775 (7)	0.0451 (3)	
F4	0.38571 (9)	0.47646 (9)	0.02241 (7)	0.0474 (3)	
O2	0.55574 (11)	0.43635 (10)	0.56371 (8)	0.0416 (3)	
F6	0.79755 (11)	0.85042 (9)	0.44537 (11)	0.0692 (5)	
F5	0.99645 (11)	0.21121 (11)	0.40542 (10)	0.0675 (4)	
F3	0.50685 (12)	0.13357 (10)	0.08038 (8)	0.0572 (4)	
O1	0.62600 (13)	0.30028 (11)	0.57362 (9)	0.0553 (4)	
N1	0.60814 (14)	0.37229 (12)	0.60903 (9)	0.0410 (4)	
C13	0.27515 (11)	0.30170 (11)	0.31982 (9)	0.0215 (3)	
C31	0.56064 (12)	0.45066 (11)	0.25939 (10)	0.0243 (3)	
C18	0.19479 (12)	0.24105 (12)	0.27736 (10)	0.0263 (3)	
H18	0.2066	0.1742	0.2672	0.032*	
C19	0.74614 (12)	0.37744 (12)	0.37632 (10)	0.0253 (3)	
C10	0.36545 (12)	−0.02184 (13)	0.48867 (11)	0.0302 (4)	
C25	0.67937 (12)	0.57296 (12)	0.38659 (11)	0.0279 (3)	
C7	0.39307 (11)	0.14240 (11)	0.40910 (9)	0.0211 (3)	
C14	0.25681 (13)	0.39942 (11)	0.33524 (10)	0.0254 (3)	
H14	0.3112	0.4413	0.3638	0.030*	
C1	0.42600 (11)	0.21343 (11)	0.26973 (9)	0.0218 (3)	
C2	0.51395 (12)	0.16120 (12)	0.28178 (10)	0.0271 (3)	
H2	0.5557	0.1437	0.3345	0.032*	
C16	0.08347 (13)	0.37408 (13)	0.26691 (10)	0.0286 (3)	
C12	0.40868 (12)	0.14764 (13)	0.49002 (10)	0.0271 (3)	
H12	0.4299	0.2081	0.5180	0.032*	
C9	0.35276 (13)	−0.03165 (12)	0.40981 (11)	0.0313 (4)	
H9	0.3354	−0.0935	0.3833	0.038*	
C24	0.74763 (14)	0.27651 (13)	0.39130 (12)	0.0374 (4)	
H24	0.6902	0.2459	0.3957	0.045*	
C11	0.39337 (13)	0.06499 (14)	0.53021 (10)	0.0321 (4)	
H11	0.4020	0.0686	0.5851	0.039*	
C20	0.83248 (14)	0.42141 (14)	0.37358 (12)	0.0350 (4)	
H20	0.8334	0.4906	0.3646	0.042*	
C17	0.09802 (13)	0.27727 (13)	0.24988 (11)	0.0301 (3)	
H17	0.0432	0.2366	0.2201	0.036*	
C32	0.46685 (12)	0.49614 (12)	0.23619 (11)	0.0297 (3)	
H32	0.4430	0.5202	0.2757	0.036*	
C8	0.36611 (12)	0.05197 (12)	0.36976 (10)	0.0266 (3)	
H8	0.3567	0.0475	0.3148	0.032*	
C15	0.15989 (13)	0.43620 (12)	0.30931 (10)	0.0292 (3)	
H15	0.1469	0.5023	0.3206	0.035*	
C6	0.36475 (13)	0.23666 (12)	0.19198 (10)	0.0279 (3)	
H6	0.3042	0.2717	0.1826	0.034*	
C5	0.39137 (15)	0.20901 (14)	0.12767 (10)	0.0349 (4)	
H5	0.3492	0.2241	0.0745	0.042*	
C30	0.72864 (15)	0.59886 (15)	0.46584 (12)	0.0389 (4)	
H30	0.7340	0.5522	0.5067	0.047*	
C26	0.66967 (14)	0.64228 (13)	0.32700 (13)	0.0371 (4)	
H26	0.6357	0.6251	0.2726	0.045*	
C3	0.54176 (14)	0.13431 (13)	0.21832 (12)	0.0337 (4)	
H3	0.6022	0.0993	0.2269	0.040*	
C36	0.59289 (13)	0.41359 (14)	0.20094 (11)	0.0323 (4)	
H36	0.6563	0.3822	0.2160	0.039*	
C33	0.40860 (13)	0.50640 (13)	0.15634 (11)	0.0331 (4)	
H33	0.3458	0.5390	0.1404	0.040*	
C34	0.44360 (14)	0.46841 (13)	0.10076 (11)	0.0326 (4)	
C4	0.47966 (15)	0.15968 (14)	0.14281 (11)	0.0357 (4)	
C23	0.83121 (16)	0.21966 (15)	0.39992 (13)	0.0458 (5)	
H23	0.8312	0.1503	0.4086	0.055*	
C35	0.53378 (15)	0.42173 (15)	0.12048 (11)	0.0372 (4)	
H35	0.5555	0.3956	0.0804	0.045*	
C22	0.91361 (15)	0.26623 (16)	0.39562 (12)	0.0424 (5)	
C29	0.77017 (16)	0.69222 (16)	0.48612 (14)	0.0475 (5)	
H29	0.8053	0.7097	0.5403	0.057*	
C21	0.91726 (15)	0.36563 (16)	0.38374 (13)	0.0427 (5)	
H21	0.9764	0.3959	0.3825	0.051*	
C27	0.70967 (16)	0.73673 (15)	0.34703 (16)	0.0477 (5)	
H27	0.7028	0.7849	0.3069	0.057*	
C28	0.75902 (15)	0.75839 (14)	0.42584 (16)	0.0471 (6)	

### (Nitrito-κ2O:O')bis[tris(4-fluorophenyl)phosphine-κP]silver(I) Atomic displacement parameters (Å^2^)

**Table:**

	*U*^11^	*U*^22^	*U*^33^	*U*^12^	*U*^13^	*U*^23^
Ag1	0.03043 (7)	0.02842 (7)	0.01960 (7)	−0.00931 (5)	0.01128 (5)	−0.00418 (4)
P1	0.02281 (19)	0.01928 (17)	0.01790 (18)	−0.00326 (13)	0.00758 (15)	−0.00146 (14)
P2	0.0260 (2)	0.02445 (19)	0.0234 (2)	−0.00463 (15)	0.01228 (16)	0.00038 (15)
F2	0.0326 (6)	0.0468 (6)	0.0390 (6)	0.0152 (5)	0.0093 (5)	0.0042 (5)
F1	0.0435 (6)	0.0404 (6)	0.0507 (7)	−0.0020 (5)	0.0163 (5)	0.0254 (5)
F4	0.0543 (7)	0.0512 (7)	0.0263 (6)	0.0068 (6)	0.0022 (5)	0.0043 (5)
O2	0.0579 (9)	0.0344 (7)	0.0262 (7)	0.0109 (6)	0.0076 (6)	−0.0034 (5)
F6	0.0642 (9)	0.0346 (6)	0.1263 (14)	−0.0266 (6)	0.0553 (9)	−0.0283 (8)
F5	0.0503 (8)	0.0639 (9)	0.0852 (11)	0.0269 (7)	0.0207 (8)	0.0008 (8)
F3	0.0779 (9)	0.0718 (9)	0.0358 (7)	0.0121 (7)	0.0375 (7)	−0.0044 (6)
O1	0.0684 (11)	0.0384 (8)	0.0392 (8)	0.0175 (7)	−0.0045 (7)	−0.0012 (6)
N1	0.0569 (11)	0.0325 (8)	0.0230 (8)	−0.0030 (7)	0.0016 (7)	−0.0008 (6)
C13	0.0266 (8)	0.0202 (7)	0.0188 (7)	−0.0011 (5)	0.0097 (6)	0.0005 (5)
C31	0.0267 (8)	0.0234 (7)	0.0251 (8)	−0.0028 (6)	0.0120 (6)	0.0010 (6)
C18	0.0278 (8)	0.0214 (7)	0.0283 (8)	−0.0011 (6)	0.0086 (7)	−0.0024 (6)
C19	0.0280 (8)	0.0267 (8)	0.0215 (8)	−0.0016 (6)	0.0095 (6)	0.0022 (6)
C10	0.0241 (8)	0.0303 (8)	0.0360 (9)	0.0018 (6)	0.0107 (7)	0.0154 (7)
C25	0.0267 (8)	0.0249 (8)	0.0364 (9)	−0.0043 (6)	0.0166 (7)	−0.0025 (7)
C7	0.0177 (7)	0.0238 (7)	0.0215 (7)	0.0005 (5)	0.0067 (6)	0.0025 (6)
C14	0.0337 (9)	0.0207 (7)	0.0220 (8)	−0.0011 (6)	0.0103 (7)	0.0001 (6)
C1	0.0264 (8)	0.0195 (7)	0.0205 (7)	−0.0055 (5)	0.0097 (6)	−0.0020 (5)
C2	0.0291 (8)	0.0282 (8)	0.0234 (8)	−0.0012 (6)	0.0090 (7)	0.0004 (6)
C16	0.0291 (8)	0.0341 (9)	0.0234 (8)	0.0091 (6)	0.0104 (7)	0.0070 (6)
C12	0.0285 (8)	0.0300 (8)	0.0222 (8)	0.0005 (6)	0.0086 (7)	0.0005 (6)
C9	0.0323 (9)	0.0222 (8)	0.0390 (10)	−0.0015 (6)	0.0125 (8)	0.0039 (7)
C24	0.0336 (9)	0.0298 (9)	0.0426 (11)	−0.0045 (7)	0.0065 (8)	0.0057 (8)
C11	0.0316 (9)	0.0424 (10)	0.0228 (8)	0.0027 (7)	0.0103 (7)	0.0094 (7)
C20	0.0361 (9)	0.0331 (9)	0.0434 (11)	0.0008 (7)	0.0234 (8)	0.0067 (8)
C17	0.0257 (8)	0.0316 (8)	0.0308 (9)	−0.0020 (6)	0.0077 (7)	−0.0018 (7)
C32	0.0291 (8)	0.0292 (8)	0.0340 (9)	−0.0014 (6)	0.0151 (7)	−0.0039 (7)
C8	0.0315 (8)	0.0236 (7)	0.0261 (8)	−0.0029 (6)	0.0118 (7)	0.0000 (6)
C15	0.0408 (10)	0.0225 (8)	0.0257 (8)	0.0070 (6)	0.0137 (7)	0.0023 (6)
C6	0.0333 (9)	0.0275 (8)	0.0231 (8)	0.0013 (6)	0.0103 (7)	0.0020 (6)
C5	0.0459 (11)	0.0395 (10)	0.0196 (8)	0.0006 (8)	0.0120 (8)	0.0018 (7)
C30	0.0460 (11)	0.0376 (10)	0.0392 (11)	−0.0112 (8)	0.0229 (9)	−0.0096 (8)
C26	0.0331 (9)	0.0325 (9)	0.0457 (11)	−0.0070 (7)	0.0142 (8)	0.0057 (8)
C3	0.0368 (10)	0.0324 (9)	0.0375 (10)	0.0020 (7)	0.0201 (8)	−0.0011 (7)
C36	0.0313 (9)	0.0402 (9)	0.0279 (9)	0.0085 (7)	0.0138 (7)	0.0052 (7)
C33	0.0290 (9)	0.0304 (9)	0.0369 (10)	0.0023 (7)	0.0084 (7)	−0.0003 (7)
C34	0.0373 (9)	0.0289 (8)	0.0263 (9)	−0.0016 (7)	0.0053 (7)	0.0036 (7)
C4	0.0521 (11)	0.0353 (9)	0.0288 (9)	−0.0026 (8)	0.0253 (9)	−0.0043 (7)
C23	0.0465 (12)	0.0302 (9)	0.0489 (12)	0.0054 (8)	0.0032 (9)	0.0036 (8)
C35	0.0441 (11)	0.0442 (10)	0.0274 (9)	0.0057 (8)	0.0178 (8)	0.0006 (8)
C22	0.0398 (11)	0.0474 (11)	0.0367 (11)	0.0157 (9)	0.0097 (9)	0.0003 (8)
C29	0.0492 (12)	0.0456 (11)	0.0566 (14)	−0.0190 (9)	0.0298 (11)	−0.0252 (10)
C21	0.0353 (10)	0.0517 (12)	0.0483 (12)	0.0034 (8)	0.0236 (9)	0.0042 (9)
C27	0.0424 (11)	0.0302 (10)	0.0741 (16)	−0.0068 (8)	0.0254 (11)	0.0097 (10)
C28	0.0373 (10)	0.0289 (9)	0.0870 (18)	−0.0129 (8)	0.0368 (11)	−0.0159 (10)

### (Nitrito-κ2O:O')bis[tris(4-fluorophenyl)phosphine-κP]silver(I) Geometric parameters (Å, º)

**Table:**

Ag1—P1	2.4457 (4)	C16—C15	1.372 (3)
Ag1—P2	2.4680 (4)	C12—H12	0.9500
Ag1—O2	2.3379 (13)	C12—C11	1.390 (2)
Ag1—O1	2.5638 (14)	C9—H9	0.9500
P1—C13	1.8206 (16)	C9—C8	1.390 (2)
P1—C7	1.8252 (15)	C24—H24	0.9500
P1—C1	1.8285 (16)	C24—C23	1.385 (3)
P2—C31	1.8197 (17)	C11—H11	0.9500
P2—C19	1.8232 (17)	C20—H20	0.9500
P2—C25	1.8220 (16)	C20—C21	1.386 (3)
F2—C16	1.3595 (19)	C17—H17	0.9500
F1—C10	1.3612 (18)	C32—H32	0.9500
F4—C34	1.356 (2)	C32—C33	1.382 (3)
O2—N1	1.239 (2)	C8—H8	0.9500
F6—C28	1.355 (2)	C15—H15	0.9500
F5—C22	1.358 (2)	C6—H6	0.9500
F3—C4	1.362 (2)	C6—C5	1.394 (2)
O1—N1	1.240 (2)	C5—H5	0.9500
C13—C18	1.395 (2)	C5—C4	1.369 (3)
C13—C14	1.394 (2)	C30—H30	0.9500
C31—C32	1.399 (2)	C30—C29	1.387 (3)
C31—C36	1.386 (2)	C26—H26	0.9500
C18—H18	0.9500	C26—C27	1.393 (3)
C18—C17	1.384 (2)	C3—H3	0.9500
C19—C24	1.389 (2)	C3—C4	1.371 (3)
C19—C20	1.391 (2)	C36—H36	0.9500
C10—C9	1.369 (3)	C36—C35	1.392 (3)
C10—C11	1.371 (3)	C33—H33	0.9500
C25—C30	1.387 (3)	C33—C34	1.370 (3)
C25—C26	1.392 (3)	C34—C35	1.366 (3)
C7—C12	1.392 (2)	C23—H23	0.9500
C7—C8	1.395 (2)	C23—C22	1.366 (3)
C14—H14	0.9500	C35—H35	0.9500
C14—C15	1.389 (2)	C22—C21	1.365 (3)
C1—C2	1.393 (2)	C29—H29	0.9500
C1—C6	1.391 (2)	C29—C28	1.369 (3)
C2—H2	0.9500	C21—H21	0.9500
C2—C3	1.387 (2)	C27—H27	0.9500
C16—C17	1.377 (2)	C27—C28	1.367 (3)
			
P1—Ag1—P2	114.924 (14)	C12—C11—H11	120.8
P1—Ag1—O1	108.89 (4)	C19—C20—H20	119.6
P2—Ag1—O1	115.63 (4)	C21—C20—C19	120.89 (17)
O2—Ag1—P1	128.18 (4)	C21—C20—H20	119.6
O2—Ag1—P2	116.74 (4)	C18—C17—H17	121.0
O2—Ag1—O1	49.80 (5)	C16—C17—C18	118.04 (16)
C13—P1—Ag1	115.64 (5)	C16—C17—H17	121.0
C13—P1—C7	102.73 (7)	C31—C32—H32	119.7
C13—P1—C1	104.54 (7)	C33—C32—C31	120.58 (16)
C7—P1—Ag1	116.30 (5)	C33—C32—H32	119.7
C7—P1—C1	103.93 (7)	C7—C8—H8	119.5
C1—P1—Ag1	112.26 (5)	C9—C8—C7	120.98 (16)
C31—P2—Ag1	109.30 (5)	C9—C8—H8	119.5
C31—P2—C19	105.73 (7)	C14—C15—H15	121.0
C31—P2—C25	102.32 (8)	C16—C15—C14	117.97 (15)
C19—P2—Ag1	115.14 (5)	C16—C15—H15	121.0
C25—P2—Ag1	120.40 (6)	C1—C6—H6	119.7
C25—P2—C19	102.36 (7)	C1—C6—C5	120.66 (16)
N1—O2—Ag1	103.97 (11)	C5—C6—H6	119.7
N1—O1—Ag1	92.63 (10)	C6—C5—H5	120.7
O2—N1—O1	113.57 (15)	C4—C5—C6	118.51 (17)
C18—C13—P1	122.04 (12)	C4—C5—H5	120.7
C14—C13—P1	118.77 (12)	C25—C30—H30	119.6
C14—C13—C18	119.17 (15)	C29—C30—C25	120.7 (2)
C32—C31—P2	116.63 (12)	C29—C30—H30	119.6
C36—C31—P2	124.56 (13)	C25—C26—H26	120.0
C36—C31—C32	118.81 (15)	C25—C26—C27	120.1 (2)
C13—C18—H18	119.6	C27—C26—H26	120.0
C17—C18—C13	120.74 (15)	C2—C3—H3	120.9
C17—C18—H18	119.6	C4—C3—C2	118.16 (17)
C24—C19—P2	118.30 (13)	C4—C3—H3	120.9
C24—C19—C20	118.37 (16)	C31—C36—H36	119.5
C20—C19—P2	123.27 (13)	C31—C36—C35	120.97 (16)
F1—C10—C9	118.21 (16)	C35—C36—H36	119.5
F1—C10—C11	118.41 (16)	C32—C33—H33	120.8
C9—C10—C11	123.39 (15)	C34—C33—C32	118.46 (16)
C30—C25—P2	117.32 (14)	C34—C33—H33	120.8
C30—C25—C26	119.51 (17)	F4—C34—C33	118.63 (16)
C26—C25—P2	123.15 (14)	F4—C34—C35	118.24 (17)
C12—C7—P1	118.05 (12)	C35—C34—C33	123.13 (17)
C12—C7—C8	119.01 (14)	F3—C4—C5	118.87 (17)
C8—C7—P1	122.88 (12)	F3—C4—C3	118.31 (18)
C13—C14—H14	119.7	C5—C4—C3	122.81 (17)
C15—C14—C13	120.70 (15)	C24—C23—H23	121.0
C15—C14—H14	119.7	C22—C23—C24	118.03 (18)
C2—C1—P1	117.51 (12)	C22—C23—H23	121.0
C6—C1—P1	123.73 (12)	C36—C35—H35	121.0
C6—C1—C2	118.56 (15)	C34—C35—C36	118.03 (17)
C1—C2—H2	119.4	C34—C35—H35	121.0
C3—C2—C1	121.28 (16)	F5—C22—C23	118.35 (19)
C3—C2—H2	119.4	F5—C22—C21	118.6 (2)
F2—C16—C17	117.91 (16)	C21—C22—C23	123.07 (19)
F2—C16—C15	118.71 (15)	C30—C29—H29	121.0
C15—C16—C17	123.37 (16)	C28—C29—C30	118.0 (2)
C7—C12—H12	119.8	C28—C29—H29	121.0
C11—C12—C7	120.44 (16)	C20—C21—H21	120.8
C11—C12—H12	119.8	C22—C21—C20	118.35 (19)
C10—C9—H9	121.1	C22—C21—H21	120.8
C10—C9—C8	117.78 (16)	C26—C27—H27	120.8
C8—C9—H9	121.1	C28—C27—C26	118.3 (2)
C19—C24—H24	119.4	C28—C27—H27	120.8
C23—C24—C19	121.24 (18)	F6—C28—C29	118.3 (2)
C23—C24—H24	119.4	F6—C28—C27	118.4 (2)
C10—C11—C12	118.34 (16)	C27—C28—C29	123.29 (18)
C10—C11—H11	120.8		
			
Ag1—P1—C13—C18	−177.10 (11)	C19—C24—C23—C22	1.9 (3)
Ag1—P1—C13—C14	1.26 (14)	C19—C20—C21—C22	0.7 (3)
Ag1—P1—C7—C12	35.13 (14)	C10—C9—C8—C7	1.0 (3)
Ag1—P1—C7—C8	−147.78 (12)	C25—P2—C31—C32	−76.35 (13)
Ag1—P1—C1—C2	63.00 (13)	C25—P2—C31—C36	102.57 (15)
Ag1—P1—C1—C6	−111.73 (13)	C25—P2—C19—C24	158.61 (15)
Ag1—P2—C31—C32	52.35 (13)	C25—P2—C19—C20	−18.40 (17)
Ag1—P2—C31—C36	−128.73 (14)	C25—C30—C29—C28	1.5 (3)
Ag1—P2—C19—C24	26.15 (16)	C25—C26—C27—C28	0.7 (3)
Ag1—P2—C19—C20	−150.86 (14)	C7—P1—C13—C18	−49.33 (15)
Ag1—P2—C25—C30	51.09 (16)	C7—P1—C13—C14	129.02 (13)
Ag1—P2—C25—C26	−130.57 (14)	C7—P1—C1—C2	−63.50 (13)
Ag1—O2—N1—O1	−1.6 (2)	C7—P1—C1—C6	121.76 (14)
Ag1—O1—N1—O2	1.38 (18)	C7—C12—C11—C10	1.8 (3)
P1—C13—C18—C17	179.14 (13)	C14—C13—C18—C17	0.8 (2)
P1—C13—C14—C15	−177.97 (12)	C1—P1—C13—C18	58.94 (14)
P1—C7—C12—C11	174.42 (13)	C1—P1—C13—C14	−122.70 (13)
P1—C7—C8—C9	−175.70 (13)	C1—P1—C7—C12	159.05 (12)
P1—C1—C2—C3	−173.68 (13)	C1—P1—C7—C8	−23.86 (15)
P1—C1—C6—C5	174.10 (13)	C1—C2—C3—C4	−0.6 (3)
P2—C31—C32—C33	177.19 (13)	C1—C6—C5—C4	−0.8 (3)
P2—C31—C36—C35	−178.48 (15)	C2—C1—C6—C5	−0.6 (2)
P2—C19—C24—C23	−179.93 (16)	C2—C3—C4—F3	179.86 (16)
P2—C19—C20—C21	178.43 (16)	C2—C3—C4—C5	−0.9 (3)
P2—C25—C30—C29	176.96 (15)	C12—C7—C8—C9	1.4 (2)
P2—C25—C26—C27	−178.00 (15)	C9—C10—C11—C12	0.6 (3)
F2—C16—C17—C18	179.26 (15)	C24—C19—C20—C21	1.4 (3)
F2—C16—C15—C14	−178.07 (15)	C24—C23—C22—F5	178.86 (19)
F1—C10—C9—C8	178.03 (15)	C24—C23—C22—C21	0.4 (3)
F1—C10—C11—C12	−179.42 (15)	C11—C10—C9—C8	−2.0 (3)
F4—C34—C35—C36	−179.96 (17)	C20—C19—C24—C23	−2.8 (3)
F5—C22—C21—C20	179.84 (19)	C17—C16—C15—C14	0.9 (3)
C13—P1—C7—C12	−92.21 (13)	C32—C31—C36—C35	0.4 (3)
C13—P1—C7—C8	84.87 (14)	C32—C33—C34—F4	178.61 (16)
C13—P1—C1—C2	−170.89 (12)	C32—C33—C34—C35	−0.7 (3)
C13—P1—C1—C6	14.37 (15)	C8—C7—C12—C11	−2.8 (2)
C13—C18—C17—C16	−1.1 (3)	C15—C16—C17—C18	0.3 (3)
C13—C14—C15—C16	−1.3 (2)	C6—C1—C2—C3	1.3 (2)
C31—P2—C19—C24	−94.61 (15)	C6—C5—C4—F3	−179.14 (16)
C31—P2—C19—C20	88.38 (16)	C6—C5—C4—C3	1.6 (3)
C31—P2—C25—C30	172.44 (14)	C30—C25—C26—C27	0.3 (3)
C31—P2—C25—C26	−9.21 (17)	C30—C29—C28—F6	178.61 (18)
C31—C32—C33—C34	1.9 (3)	C30—C29—C28—C27	−0.4 (3)
C31—C36—C35—C34	0.8 (3)	C26—C25—C30—C29	−1.4 (3)
C18—C13—C14—C15	0.4 (2)	C26—C27—C28—F6	−179.71 (18)
C19—P2—C31—C32	176.84 (12)	C26—C27—C28—C29	−0.7 (3)
C19—P2—C31—C36	−4.24 (17)	C36—C31—C32—C33	−1.8 (2)
C19—P2—C25—C30	−78.17 (15)	C33—C34—C35—C36	−0.7 (3)
C19—P2—C25—C26	100.17 (16)	C23—C22—C21—C20	−1.7 (3)

### (Nitrito-κ2O:O')bis[tris(4-fluorophenyl)phosphine-κP]silver(I) Hydrogen-bond geometry (Å, º)

**Table:**

*D*—H···*A*	*D*—H	H···*A*	*D*···*A*	*D*—H···*A*
C2—H2···F1	0.95	2.42	3.367 (2)	175
C35—H35···F6	0.95	2.40	3.203 (3)	143
